# Opportunities for Increased Nitrogen Use Efficiency in Wheat for Forage Use

**DOI:** 10.3390/plants9121738

**Published:** 2020-12-09

**Authors:** Nirmal Sharma, Raquel Schneider-Canny, Konstantin Chekhovskiy, Soonil Kwon, Malay C. Saha

**Affiliations:** 1 Grass Genomics, Noble Research Institute, LLC, 2510 Sam Noble Parkway, Ardmore, OK 73401, USA; nsharma@noble.org (N.S.); rstri@hotmail.com (R.S.-C.); kchekhovskiy@noble.org (K.C.); 2 Powell Research and Extension Center, University of Wyoming, 747 Road 9, Powell, WY 82435, USA; 3 Scientific Computing, Noble Research Institute, LLC, 2510 Sam Noble Parkway, Ardmore, OK 73401, USA; skwon@noble.org

**Keywords:** wheat, forage, biomass, nitrogen use efficiency (NUE), nitrogen nutrition index (NNI)

## Abstract

Wheat is a major cool-season forage crop in the southern United States. The objective of this study is to understand the effect of nitrogen (N) fertilization on wheat biomass yield, quality, nitrogen use efficiency (NUE), and nitrogen nutrition index (NNI). The experiments were conducted in a greenhouse and a hoop house in a split-plot design, with three replications. Twenty wheat cultivars/lines were evaluated at four N rates (0, 75, 150, and 300 mg N.kg^−1^ soil) in the greenhouse and (0, 50, 100, and 200 mg N.kg^−1^ soil) in the hoop house. In general, high-NUE lines had lower crude protein content than the low-NUE lines. None of the cultivars/lines reached a plateau for biomass production or crude protein at the highest N rate. The line × N rate interaction for NUE was not significant in the greenhouse (*p* = 0.854) but was highly significant in the hoop house (*p <* 0.001). NNI had a negative correlation with NUE and biomass. NUE had strong positive correlations with shoot biomass and total biomass but low to moderate correlations with root biomass. NUE also had a strong positive correlation with N uptake efficiency. Lines with high NUE can be used in breeding programs to enhance NUE in wheat for forage use.

## 1. Introduction

Wheat is a unique and versatile dual-purpose (grain and forage use) crop in the southern Great Plains of the United States [1]. It is a good choice for winter pasture production in the region due to its high nutritive value and biomass potential [2]. In Oklahoma, 75% of the wheat planted every year is grazed on for at least part of the growing season (9% forage, 66% forage and grain, 25% grain only) [3]. Wheat supplies high-quality forage during late fall, winter, and early spring when other common forage species are not productive [4,5]. Adequate soil fertility is crucial for the fast and strong establishment of wheat in the field [4]. Farmers usually apply 18% more nitrogen (N) in wheat fields utilized for forage and grain than for grain only [3]. The recommended N fertilizer rate in wheat pastures in Oklahoma is 33 kg.ha^−1^ of N for every 1.1 Mg.ha^−1^ of forage yield. Wheat forage production in Oklahoma varies from 2.2 to 9.0 Mg.ha^−1^ [6]. About 45 kg N.ha^−1^ is applied to aid early establishment, followed by additional fertilization of 100 kg N.ha^−1^. To meet the forage production goal, supplemental N should be applied before 1st March [6]. As in other grasses, wheat responds to N more than any other nutrient. Consequently, N is usually the most limiting nutrient associated with wheat forage production [7].

Recently, concern over the harmful effects of N fertilization in the agricultural ecosystem, especially in the microbial community, has increased significantly [8]. The extravagant use of this nutrient after the Green Revolution is becoming ecologically unsustainable, and it is imprudent to talk about N requirements without considering ways to optimize N use efficiency (NUE) [9]. It has been estimated that 50% or less of the N applied to cropland is recovered by cereals, and this percentage decreases as the N fertilizer rate increases [10,11]. Globally, up to 64% (an average of 18%) of applied N is lost via NH_3_ volatilization [12]. In wheat, 7.7% to 59.4% of plant N is lost between anthesis and 14 days postanthesis. In forage-only systems, gaseous N loss is lower because the plants are utilized before flowering, which can lead to improved NUE [13]. Nitrate leaching is a major worldwide cause of groundwater N pollution [14]. Actual plant N status in crops can be determined by the N nutrition index (NNI), a basic tool to quantify the level of both N deficiency and excess consumption of a specific crop [15]. Accomplishing a balance between N supply and crop demand, without excess or deficiency, is the key to optimizing trade-offs amongst yield, profit, and environmental protection in any agricultural system worldwide [16].

N losses by leaching and denitrification can be decreased by breeding crop varieties that are more efficient at capturing soil N during the entire growing season [17]. NUE is a product of N uptake efficiency (NUpE) and N utilization efficiency (NUtE) [18]. Genetic variability for both NUpE and NUtE has been documented for a large number of crops [19]. This process is governed by the interaction of multiple genetic and environmental factors [17]. NUpE is a function of root biomass, the morphological ability to explore regions with abundant nutrients, and the physiological capacity for nutrient uptake [20]. Increasing the recovery of N from fertilizer is a quick way of improving agricultural NUE in crops [21]. NUtE, on the other hand, consists of the yield of the crop per unit of N acquired by the plant [22]. When plants grow in limited N conditions, NUtE is an essential process that determines most of the variation in NUE [19]. A combination of favorable alleles associated with both NUpE and NUtE is important when breeding for NUE. Increased NUpE and NUtE may allow growers to maximize yield with moderate N fertilization instead of the traditional high rate of N application [23]. Most wheat NUE and NNI research is focused on grain production [24,25,26,27,28], and information on NUE and NNI in wheat for forage use is limited [29]. 

Considering the importance of wheat pastures in the southern USA, we initiated this project to explore 20 wheat varieties and breeding lines with potential forage use for NUE and NNI in greenhouse and hoop house experiments. Soil collected from southern Oklahoma fields were used in these experiments. The greenhouse experiment was conducted under controlled temperature and photoperiod, with four N treatments (0, 75, 150, and 300 mg N.kg^−1^ soil) in 2.5 L vases. The second experiment was established in a hoop house under natural temperature, with four N treatments (0, 50, 100, and 200 mg N.kg^−1^ soil) in 18.9 L buckets. Additionally, six high NUE lines from the hoop house experiment were also evaluated for root biomass and related traits. Wheat lines with enhanced NUE identified in this study can be used in breeding programs to develop improved cultivars that can minimize N fertilizer use and become environmental-friendly.

## 2. Results and Discussion

### 2.1. Greenhouse and Hoop House Evaluations

Statistically significant differences were detected between N rates and lines for all variables evaluated in both greenhouse and hoop house experiments, with the exception of NUpE (Table 1). Wheat lines were similar for NUpE in the greenhouse (*p* = 0.458) and N rates in the hoop house (*p* = 0.577). However, NUtE was the only variable with a significant N rate × line interaction in the greenhouse. In the hoop house, interactions were significant for biomass, NUpE, and NUE (Table 1). 

The R^2^ values for the utilized model were >80% for the majority of variables. NUpE was an exception, with the lowest value in the greenhouse (53.7%). NUpE also had the lowest R^2^ value in the hoop house (75%), but it was considerably higher than that in the greenhouse (Table 2). In general, the R^2^ values in the hoop house were higher for all variables, which indicates more precise results than those in the greenhouse. Except for NUpE, the rest of the variables (biomass, crude protein (CP), NUtE, and NUE) showed high R^2^ values (>80%), which indicate that most of the variation can be explained by the statistical model used. NUpE depends on the root’s ability to uptake nutrients and is directly related to root biomass, morphology, and physiology [20]. The use of small containers (2.5 L vases) in the greenhouse could be the reason for nonsignificant differences among lines and the N rate × line interaction and lower R^2^ values for NUpE. The smaller container may have caused limited root development in the lines with higher root growth potential.

Schneider-Canny et al. [30] also reported the limitation of smaller pots in bermudagrass. Since higher plant mass per pot volume decreases plant growth and can affect differences between treatments, proper pot size is important [31]. Thus, in the hoop house experiment, larger vases (18.9 L) were used, and more significant results were obtained due to fewer limitations for shoot and root development.

### 2.2. Biomass Production

**Figure 1:**
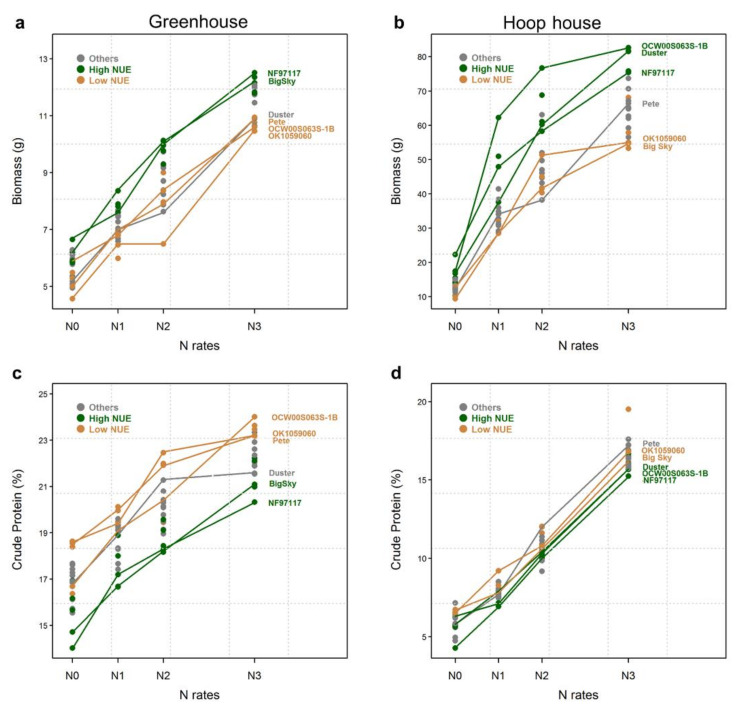
Biomass (**a**,**b**) and crude protein (**c**,**d**) of 20 winter wheat cultivars/lines evaluated in greenhouse (left panel) and hoop house (right panel) under four different N rates. Green and orange points correspond to the lines with the highest and lowest NUEs, respectively, in each environment, which were also evaluated for root traits. Grey points are all the other lines.

N fertilization increased biomass production at every N increment for all the wheat lines in the greenhouse (Figure 1a). Big Sky and NF97117 had some of the highest biomass production across N rates in the greenhouse. The variation between the lines within the treatment is similar (<3 g at N_0_, N_1_, and N_3_). A bigger variation was observed at N_2_, where the line OK1059060 had no increments in biomass compared to N_1_. However, none of the lines seemed to reach a plateau for biomass production with the N rates utilized in the greenhouse (Figure 1a). On the other hand, there was a small variation among the lines for biomass at N_0_ compared to the wide variation at N_1_, N_2_, and N_3_ in the hoop house. The OCW00S063S-1B line had the highest biomass production at the N^+^ rates, together with Duster at N_3_.

The NF97117 line had high biomass at N_0_ and did not differentiate from the top two lines at N_3_ (Figure 1b, Table 1). However, Big Sky had one of the lowest biomasses for all N rates in the hoop house. As in the greenhouse, none of the lines in the hoop house seemed to reach a plateau for biomass production with N rates. Overall, for all N rates in both environments, the lines with high NUE produced the highest biomass (Figure 1a,b).

### 2.3. Crude Protein Content

The increment of N also significantly (*p* < 0.001) increased the protein content of most of the wheat lines, with increasing N rates in both the greenhouse and the hoop house (Figure 1c,d, Table 1). In general, high-NUE lines had a lower CP content than the low-NUE lines in both experiments (Figure 1c,d). In the hoop house, the CP at N_0_ was very low (<7.5%) and all lines sharply increased the CP content with added N. As with biomass production, CP content did not reach a plateau at N_3_ in the hoop house. The lines did not differ in CP content under different N rates. The line × N rate interaction was also nonsignificant in both environments. It was also reported that high-NUE lines showed lower CP content compared to low-NUE lines in wheat [32] and bermudagrass [30]. However, both biomass dry matter and CP concentration in the forage have vital roles in determining feed value [33]. Thus, a reasonable compromise between forage quantity and quality needs to be considered in breeding programs.

### 2.4. Nitrogen Uptake Efficiency

**Figure 2:**
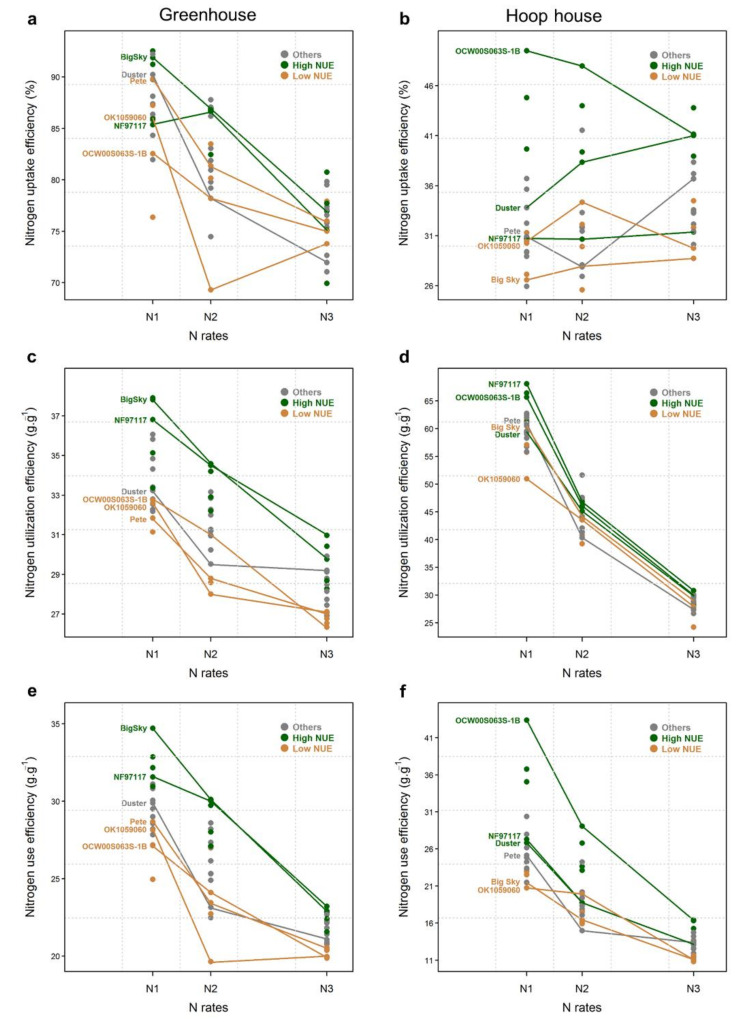
Nitrogen uptake efficiency (**a**,**b**), nitrogen utilization efficiency (**c**,**d**), and nitrogen use efficiency (**e**,**f**) of 20 wheat lines evaluated in greenhouse (left panel) and hoop house (right panel) under three different N rates. Green and orange points correspond to the six lines with the highest and lowest NUEs, respectively, in each environment, which were also evaluated for root traits. Grey points are all the other lines.

In the greenhouse, even though the addition of N significantly decreased NUpE (Table 1), the values were considerably higher than those in the hoop house at all N rates (Figure 2a,b). At N_1_ and N_2,_ most of the lines had NUpE above 80%, and at N_3_, it was between 70–80%. Well-balanced N input and output generally have 80–90% NUpE [34]. Thus, at N rates above 150 mg N.kg^−1^ soil, plants in small pots with restricted root growth may not be able to uptake the added N efficiently. Distinct differences between high and low-NUE lines for NUpE were not evident, and there were no significant differences among lines at all N rates (Figure 2a, Table 1). In the hoop house, NUpE was very low, with maximum values below 50% and no differences among N rates (Figure 2b, Table 1). The reason for lower NUpE may be the soil of the hoop house, which had very low organic matter (0.6%) content, indicating that the low potential of soil to supply N to plants as organic matter is a good index of N availability. NUpE values below 70% can cause risk of N losses, and very high and very low NUpE values can be the cause of unsustainable crop production [34]. The OCW00S063S-IB line had the highest NUpE at N_1_ and N_2_ but decreased at N_3_, while NF97117 showed almost the same NUpE at all N rates. In general, NUpE declined with increasing N rates in bread wheat [32]. Interestingly, Duster sharply increased its NUpE at higher N rates. 

### 2.5. Nitrogen Utilization Efficiency

Analysis of variance for NUtE indicated a statistically significant influence (*p* = 0.05) in the greenhouse but no influence (*p* = 0.241) in the hoop house for N rates and lines. The variation among the lines was high for NUtE in the greenhouse but low in the hoop house. NUtE declined in both environments with an increasing rate of N application. Similarly, a decrease in NUtE with an increased N rate was found in bermudagrass and bread wheat [30,32]. Low NUtE at higher N rates indicates a resistance mechanism toward high N. The NF97117 line performed better in both environments (Figure 2c,d). Additionally, this line also showed higher yield potential in different environments (Figure 1a,b), indicating that NF97117 can be advantageous from a breeding perspective and can be utilized as a potential parent in future breeding programs.

### 2.6. Nitrogen Use Efficiency

NUE decreased as more N is applied, and the variation between lines was smaller at higher N rates. Limón-Ortega et al. [35] also observed that NUE in wheat decreased as the N rate increased. Surprisingly, Big Sky had high NUE in the greenhouse and low NUE in the hoop house, while OCW00S063S-IB ranked first in all N rates in the hoop house and had poor NUE in the greenhouse (Figure 2e,f). It is possible that the roots of OCW00S063S-IB were restricted in small pots in the greenhouse but were able to grow well in large containers in the hoop house, and the roots were able to uptake more N (Figure 2a,b). NF97117 consistently showed higher NUE at both evaluations. NUE showed statistically nonsignificant line × N rate interaction (*p* = 0.854) in the greenhouse, indicating that N use-efficient lines are the same at low and high N rates. In a related approach to NUE, lines that responded well to low N-inputs also performed well with high-N inputs in wheat [24]. On the other hand, statistically highly significant differences (*p* < 0.001) were obtained in the line × N rate interaction for NUE in the hoop house, indicating that N use-efficient lines are not the same at low and high N rates. Line × N rate interactions were also reported in maize (*Zea mays* L.) [36] and rice (*Oryza sativa* L.) [37,38]. Lines with higher NUE could play an important role in sustainable agricultural systems by improving crop yields, decreasing the cost of production, and maintaining environmental quality [11]. Lines performing at higher NUE in different environments can play a significant role in future wheat breeding programs for improving NUE.

### 2.7. Nitrogen Nutrition Index

**Figure 3:**
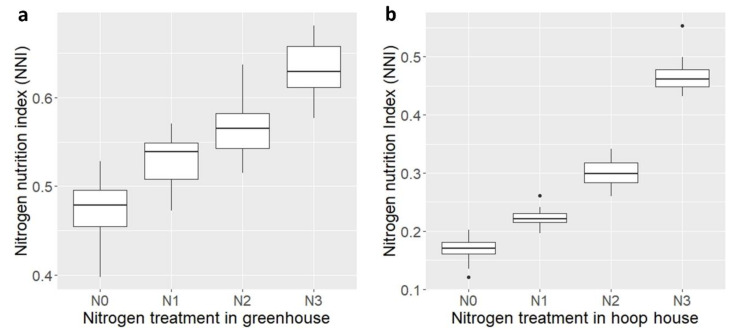
Boxplot of four different nitrogen treatments for the nitrogen nutrition index (NNI) of 20 wheat lines evaluated in greenhouse (**a**) and hoop house (**b**).

**Figure 4:**
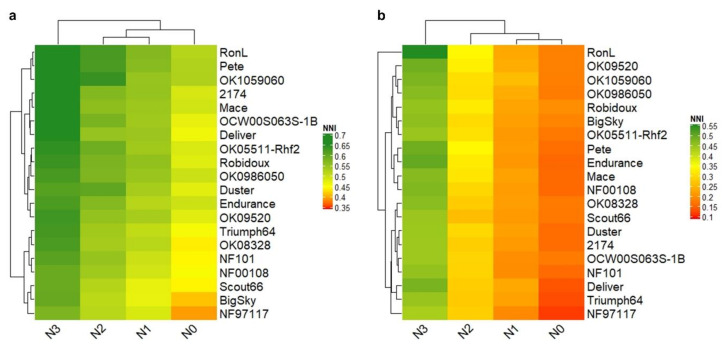
Heatmap among four different nitrogen treatments for the nitrogen nutrition index (NNI) of 20 wheat lines evaluated in greenhouse (**a**) and hoop house (**b**).

Nitrogen nutrition indices varied from 0.39 to 0.68 in the greenhouse and 0.12 to 0.55 in the hoop house (Figure 3). Values of NNI that are greater or equal to 1.0 indicate surplus N supply to the crop, whereas values less than 1.0 suggest N deficiency. In this study, NNI was significantly affected by the fertilization level (Figure 3, Table 1) in both greenhouse and hoop house. NNI values of ≤0.68 or 0.55 in the greenhouse and hoop house experiments, respectively, may justify the reason that none of the wheat lines reached a plateau for biomass production or CP across N rates. Generally, NNI is used as an indicator of N stress in maize and wheat, and, usually, NNI values increase with the addition of N fertilizer [39,40]. Typically, high-NUE lines showed lower NNI than the low-NUE lines in both greenhouse and hoop house experiments (Figure 2e,f and Figure 4a,b). However, NNI can be effectively estimated in field-grown plants at the plot scale [15]. NNI calculated for plants grown in pots gave a rough indication of the effect of the different levels of N treatments [27]. 

### 2.8. Root Evaluation in Hoop House

**Figure 5:**
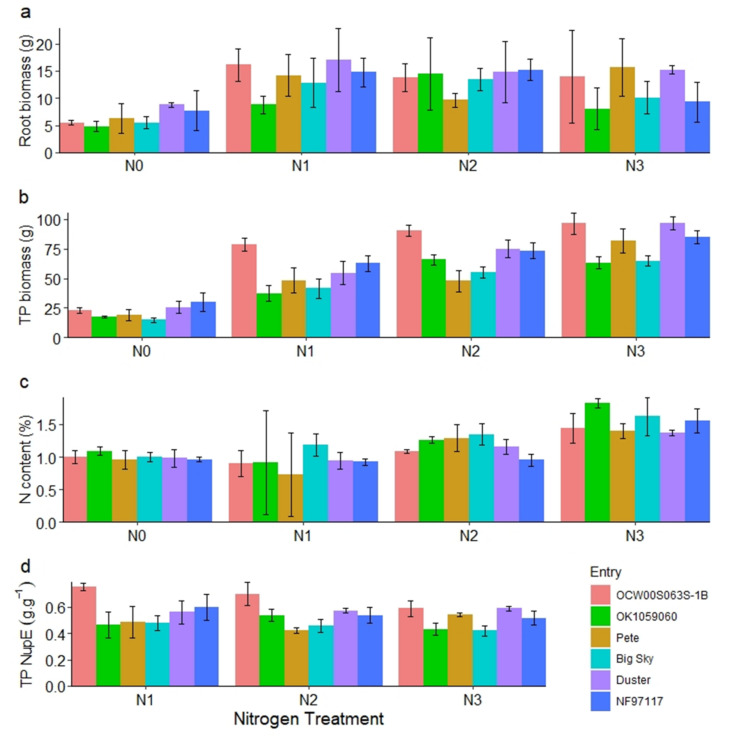
Root biomass (**a**), total plant biomass (**b**), N content (**c**), and total plant N uptake efficiency (**d**) of the six wheat lines evaluated in hoop house under three different N rates.

We evaluated the root biomass of selected lines due to discrepancies in NUE of some lines between greenhouse and hoop house experiments. There were significant differences among the six wheat lines for root biomass, TP biomass, and TP NUpE, but no statistical differences for root N content (*p* = 0.161). The N rates affected all variables except TP NUpE (*p* = 0.229). The N rate × line interaction was statistically significant for TP biomass and TP NUpE only (Table 1). TP biomass obtained the highest R^2^ value (97.3%). The smallest R^2^ was for root N (69.7%), followed by root biomass (76.3%) and TP NUpE (81%; Table 2). Root biomass of all the lines increased considerably between N_0_ and N_1_ (Figure 5a). However, root biomass tended to be stabilized at higher N rates. Duster had the highest root biomass at N_0_ and N_1_, and at N_3_, Pete, Duster, and OCW00S063S-1B had the highest root biomass. Total plant (TP) biomass gradually increased for all six lines except OK109060. The lines Pete, Duster, and OCW00S063S-1B had the highest total plant biomass at N_3_, like root biomass (Figure 5b). N content of the lines was similar within the N treatments (Figure 5c). Like root biomass and TP biomass, OCW00S063S-1B, Duster, and Pete showed higher TP NUpE (Figure 5d). Higher root densities showed higher NUpE in a winter wheat cultivar, as reported by Rasmussen et al. [41].

### 2.9. Relationship among Traits

Correlation analysis revealed that NNI was negatively associated with NUE, NUpE, NUtE, and biomass in both the greenhouse and the hoop house. However, the relationship between NNI and NUpE was not statistically significant (Figure S1). In this study, as in most previous N fertilization studies, NUE was negatively correlated with NNI [42,43,44]. NUE showed significant moderate and strong positive correlations with N uptake efficiency in the greenhouse and the hoop house, respectively. Van Sanford and MacKown [45] also reported a positive correlation between NUE and NUpE in winter wheat.

Among the six wheat lines evaluated in the hoop house, NUE showed a perfect positive relationship with shoot biomass—very high with NUpE, TP biomass, and TP NUpE and moderate with NUtE and root biomass at all N treatments (Table 3). A poor positive correlation was observed between NUtE and NUpE, which tended to decrease as more N was applied. As expected, shoot N content, which is a part of CP, showed a negative correlation with biomass, NUtE, NUpE, and NUE [24,32]. Furthermore, NUpE showed a positive correlation with NUtE.

## 3. Materials and Methods

### 3.1. Plant Materials and Soil

The germplasm consisted of 20 winter wheat accessions that included 12 cultivars and 8 experimental lines (Table 4). The accessions were primarily selected for specific characteristics, i.e., disease resistance [46,47,48,49], grazing tolerance [50], forage production [51], grain production, or dual-purpose use [52,53,54]. However, in this study, the germplasm was evaluated by considering forage-purpose production.

Soil for the greenhouse experiment was collected from a field in Ardmore, OK, which was inherently low in N content. Soil analysis revealed that it had 5.7 pH, 1.1% organic matter, and 4 mg NO_3_ kg^−1^ soil. Prior to the utilization, the soil was sterilized to eliminate microorganisms and deactivate weed seeds. As forages are generally grown on marginal soil, for a second experiment, sandy-loam soil was collected from the Red River farm, Burneyville, OK. The soil characteristics were estimated as 7.9 pH, 0.6% organic matter, and 23 mg NO_3_ kg^−1^ soil.

### 3.2. Greenhouse and Hoop House Evaluations

Seeds from the 20 wheat lines were placed in Petri dishes for germination. After three days, 60 germinated seeds from each line were transplanted to cell trays (25.4 × 25.4 mm) filled with Metro-Mix 830 (Sun Gro Horticulture, Agawam, MA, USA) and allowed to grow for two weeks in the greenhouse. The seedlings were finally transplanted to 2.5 L vases (165 × 150 mm) containing 1.5 kg of 4:1 mixture of Metro-Mix 830 in a density of four plants per vase. The vases were placed in plastic saucers (140 × 80 mm) containing 3 cm of perlite at the bottom. The perlite helped the roots reach the extra solution leached from the vases. Each vase was composed as an experimental unit and arranged in a split-plot design with three replications, having N rates in the main plot and wheat lines in the subplots. A nutrient solution (without N), adjusted according to the initial soil sample analysis, was added to all the plants the following week. Urea was used as a source of N, and the four rates consisted of 0 (N_0_), 75 (N_1_), 150 (N_2_), and 300 (N_3_) mg N.kg^−1^ soil. The treatments in which N was added are referred to as N_+_ treatments. The growth conditions were set at 32.2 °C in the day and 21.1 °C at night, and 16 h of light. To simulate a forage production system, plants were clipped at 5 cm when most of the plants reached 25 cm. Fresh weights were taken, and samples were dried at 75 °C for 72 h to obtain DM. The plants were clipped four times with an interval of ~30 days at the tillering stage. Fertilizer was added in two split applications after the first and second clippings. The total N and CP content of the harvested samples was estimated through a FOSS 6500 near-infrared reflectance spectroscope (Foss Nirsystems, Silver Spring, MD, USA).

We realized the limitations of small containers and the controlled environment in our first experiment. We decided to conduct a second experiment with large vases (18.9 L, 36.8 × 26.0 cm) under ambient temperature without the interference of rainwater. In the second experiment, a plastic-covered hoop house was used to evaluate the NUE of the same plant materials mentioned earlier. In this study, two buckets were utilized, placing one bucket inside the other. The inside bucket contained six holes to allow the passage of leached nutrient solution and roots. The outside bucket kept the solution leached through the soil and made it available to the plant roots. The buckets were filled with 18 kg of the soil mixture described above. One-week-old seedlings were transplanted to the buckets in a density of six plants per bucket. Plants were arranged in the same experimental design used in the greenhouse, using 20 lines, four N rates, and three replications. Weeds were manually removed each week. Each bucket of plants was watered manually, as needed, to avoid overwatering. Four N treatments, 0 (N_0_), 50 (N1), 100 (N2), and 200 (N3) mg N. kg^−1^ soil, were applied as urea in two equal applications. The plants were clipped two times, with an interval of ~30 days at the tillering stage to evaluate forage production potential. The data for shoot biomass production and N content were obtained following the protocol described in the greenhouse experiment. 

After the last clipping, the roots from the six lines (Big Sky, Duster, NF97117, OCW00S063S-1B, OK1059060, and Pete), growing in all N rates and replications, were also collected to evaluate root biomass and N content. The roots were soaked, washed, and rinsed with water to remove soil. After drying at room temperature, each root sample was checked for the presence of leftover soil and small pebbles. The samples were then oven-dried to obtain DM values, and the total N content was determined by the high-temperature combustion method (LECO Corp. CHN-600 elemental analyzer, Saint Joseph, MI, at Texas A&M University Soil, Water and Forage Testing Laboratory, College Station, TX, USA). TP biomass was obtained by adding shoot and root dry biomass. TP N content was also calculated by adding shoot and root N content.

### 3.3. Calculating NUE and NNI

Based on the shoot data, the NUE of the lines was calculated according to Moll et al. [18] as biomass produced per unit of N available in the soil, with minor modifications. NUE is the product of NUpE and NUtE, where NUpE is the capacity of plant roots to acquire N from the soil. Additionally, NUtE is plant biomass productivity per unit of N uptake (Nup), calculated following the protocol of Hawkesford [22]. Nup is N contained in the plant biomass. The amount of N available (Nav) in the soil is quantified as the sum of N from fertilizer applied (Nf) plus the N uptake by aboveground plant tissues (Nt) in pots with no N fertilizer used (N_0_) [30]. The formulas used are the following:

Nav (g) = Nf (g) + Nt (g)(1)

NUpE (%) = (Nup g/Nav g) × 100(2)

NUtE (g.g^−1^) = biomass (g)/Nup (g) and, NUE (g.g^−1^) = biomass (g)/Nav (g) (3)

Utilizing the data obtained from the roots, total plant ability to uptake N (TP NUpE) was also calculated, as follows:

TP NUpE = (TP Nup g/Nav g) × 100(4)

The NNI is a ratio between the N content of a crop to its critical content, indicating the minimum N required for the maximum biomass production. The NNI analysis is based on the parameters of the N dilution curve [15], and, for wheat, critical N concentration was reported by Justes et al. (1997) [40]. In this study, NNI was used as a simple index, reflecting the N nutrition of the plants for each N treatment in pot experiments. NNI was calculated by assuming a critical shoot N concentration of 5.64% at Zadoks tillering growth stage 26 [27,55]. NNI of the crop at each harvesting date was determined by dividing the actual plant N concentration (N_a_) of the shoot biomass by the critical N concentration (N_c_) [56].

### 3.4. Statistical Analysis

The data sets from the greenhouse and hoop house experiments were analyzed separately. The response variables, shoot biomass, and shoot CP were analyzed by combining N_0_ and N^+^ traits and also with N rates only in order to better visualize the effect of N fertilization and its interactions. The response variables NUE, NUpE, and NUtE, as well as TP NUpE, were calculated for N^+^ rates. For all data sets, a split-plot in completely randomized design (CRD) was employed, where N rates and lines were applied to the main plot and subplots, respectively. The replicates were treated as random factors. Analyses of variances were performed with SAS 9.3 (SAS Institute Inc., Cary, NC, USA). Significance was declared if *p*-values were less than 0.05 significance level. The minimum LSD statistics were approximately calculated for each trait since all sample sizes were not the same for all lines. The R^2^ of the model was calculated for each response variable. Pearson’s pairwise correlation coefficients between different traits were calculated using the pairs.panels of psych [57] package in R. Heatmaps, and boxplots of line responses within the N rates for the greenhouse and the hoop house were obtained from the ComplexHeatmap [58] and ggplot2 [59] packages in R, respectively. Pearson’s correlations among response variables for the six lines were performed using SAS 9.3.

## 4. Conclusions

Wheat is a good source of quality forage, especially during fall and winter, when warm-season grasses are not able to grow due to cold temperatures. The larger vases used to grow wheat for studies seemed to have less limitation for shoot and root development, offering more suitable conditions for NUE studies in wheat. The wheat accessions presented high variability for NUE. In general, NUE decreased with an increase in N rate. On the other hand, NNI increased with the rising of the N rate. NUE is highly positively correlated with wheat biomass production. However, a significant negative relationship was observed between NUE and CP content. Thus, a reasonable compromise between forage yield and quality needs to be established in wheat breeding programs that are focused on forage use. Accessions that are consistently identified with high NUE in both greenhouse and hoop house experiments are sensible parental materials for NUE improvement programs.

## Figures and Tables

**Table 1:** *F*-test of fixed effects for biomass, crude protein, nitrogen uptake efficiency, nitrogen utilization efficiency, and nitrogen use efficiency in greenhouse and hoop house. *F*-test of fixed effects for root biomass, root N, total plant biomass, and total plant nitrogen uptake efficiency for six wheat lines grown in the hoop house.

Variables	N Rate	Line	N Rate × Lines
	*All Lines in Greenhouse (p > F)*		
Biomass (all N rates)	0.001	<0.001	0.965
Crude protein (all N rates)	<0.001	<0.001	0.168
Nitrogen nutrition index (all N rates)	<0.001	<0.001	0.267
Biomass (N^+^)	0.008	<0.001	0.934
Crude protein (N^+^)	<0.001	<0.001	0.089
Nitrogen uptake efficiency	0.005	0.458	0.978
Nitrogen utilization efficiency	0.005	<0.001	0.050
Nitrogen use efficiency	0.009	<0.001	0.854
Nitrogen nutrition index (N^+^)	<0.001	<0.001	0.099
	*All lines in hoop house (p > F)*		
Biomass (all N rates)	<0.001	<0.001	<0.001
Crude protein (all N rates)	<0.001	<0.001	0.229
Nitrogen nutrition index (all N rates)	<0.001	<0.001	0.330
Biomass (N^+^)	0.0005	<0.001	0.005
Crude protein (N^+^)	<0.001	<0.001	0.449
Nitrogen uptake efficiency	0.577	<0.001	<0.001
Nitrogen utilization efficiency	<0.001	<0.001	0.241
Nitrogen use efficiency	0.023	<0.001	<0.001
Nitrogen nutrition index (N^+^)	<0.001	<0.001	0.489
	*Six lines in hoop house (p > F)*		
Root biomass	0.002	0.013	0.188
Root N	0.002	0.161	0.806
TP biomass	<0.001	<0.001	<0.001
TP nitrogen uptake efficiency	0.229	<0.001	0.042

**Table 2:** R^2^ values (%) of the split-plot in CRD statistical model for variables of all wheat lines evaluated in greenhouse and hoop house for biomass, crude protein, nitrogen nutrition index, nitrogen uptake efficiency, nitrogen utilization efficiency, and nitrogen use efficiency. R^2^ values (%) of the split-plot in CRD statistical model for root-related traits of selected six wheat lines evaluated in the hoop house.

Variables	Greenhouse	Hoop House
Biomass (all N rates)	94.9	96.5
Crude protein (all N rates)	94.9	96.7
Nitrogen nutrition index (all N rates)	94.3	96.4
Biomass (N^+^)	88.1	92.0
Crude protein (N^+^)	94.1	95.9
Nitrogen uptake efficiency	53.7	75.0
Nitrogen utilization efficiency	94.2	95.1
Nitrogen use efficiency	82.4	90.4
Nitrogen nutrition index (N^+^)	93.8	95.6
	*Six lines in hoop house*	
Root biomass		76.3
Root N		69.7
TP biomass		97.3
TP nitrogen uptake efficiency		81.0

**Table 3:** Pearson’s correlation among shoot, root, and TP variables related to nitrogen use efficiencies of six wheat lines grown under four different N rates in hoop house.

	N Rate	Shoot N (%)	Root Biomass	Root N (%)	TP Biomass	TP NUpE	NUtE	NUpE	NUE
	N_0_	0.64 *	0.55 *	0.34	0.96 *	-	-	-	-
**Shoot biomass**	N1	−0.67 *	0.53 *	−0.17	0.97 *	0.90 *	0.67 *	0.96 *	1.00 *
	N2	−0.50 *	0.20	−0.51 *	0.97 *	0.89 *	0.50 *	0.92 *	1.00 *
	N3	−0.52 *	0.38	−0.55 *	0.95 *	0.86 *	0.52 *	0.92 *	1.00 *
	N_0_		−0.32	−0.38	−0.60 *	-	-	-	-
**Shoot N (%)**	N1		−0.65 *	0.10	−0.73 *	−0.51 *	−0.99 *	−0.41	−0.64 *
	N2		−0.25	0.42	−0.52 *	−0.16	−0.98 *	−0.12	−0.51 *
	N3		0.18	0.25	−0.36	−0.06	−1.00 *	−0.14	−0.51 *
	N_0_			0.25	0.76 *	-	-	-	-
**Root biomass**	N1			−0.02	0.71 *	0.59 *	0.63 *	0.44	0.56 *
	N2			−0.34	0.43	0.35	0.22	0.14	0.19
	N3			−0.47 *	0.65 *	0.70 *	−0.19	0.54 *	0.40
	N_0_				0.35	-	-	-	-
**Root N (%)**	N1				−0.15 *	0.16	−0.10	−0.19	−0.19
	N2				−0.55 *	−0.38	−0.39	−0.41	−0.48 *
	N3				−0.61 *	−0.50 *	−0.26	−0.59 *	−0.62 *
	N_0_					-	-	-	-
**TP biomass**	N1					0.90 *	0.72 *	0.91 *	0.97 *
	N2					0.91 *	0.52 *	0.89 *	0.97 *
	N3					0.95 *	0.36	0.94 *	0.96 *
**TP NUpE**	N1						0.50 *	0.91 *	0.89 *
	N2						0.14	0.97 *	0.89 *
	N3						0.06	0.98 *	0.87 **
**NUtE**	N1							0.40	0.64 *
	N2							0.11	0.51 *
	N3							0.14	0.51 *
**NUpE**	N1								0.96 *
	N2								0.91 *
	N3								0.92 *

* Significant at *p* < 0.05, ** Significant at *p* < 0.01.

**Table 4:** Wheat cultivars/lines evaluated in greenhouse and hoop house for nitrogen use efficiency (NUE).

Wheat Lines	GRIN Accession	Improvement Status	Trait
2174	-	Cultivar	Dual-purpose
Big Sky	PI 619166	Cultivar	Grain production
Deliver	PI 639232	Cultivar	Dual-purpose
Duster	PI 644016	Cultivar	Dual-purpose
Endurance	PI 639233	Cultivar	Grazing-tolerant, disease-resistant
Mace	PI 651043	Cultivar	Disease-resistant
NF00108	-	Experimental line	
NF101	-	Cultivar	Forage
NF97117	-	Experimental line	
OCW00S063S-1B	-	Experimental line	
OK05511-Rhf2	-	Experimental line	
OK08328	-	Experimental line	
OK09520	-	Experimental line	
OK0986050	-	Experimental line	
OK1059060	-	Experimental line	
Pete	PI 656844	Cultivar	Dual-purpose
Robidoux	PI 659690	Cultivar	Disease-resistant
Ron-L	-	Cultivar	Disease-resistant
Scout 66	CItr 13996	Cultivar	Disease-resistant
Triumph 64	CItr 13679	Cultivar	Disease-resistant

## Supplementary Materials

The following are available online at https://www.mdpi.com/2223-7747/9/12/1738/s1. Figure S1: Correlation among biomass, NUtE, NUpE, NUE, and NNI of 20 wheat cultivars/lines evaluated in greenhouse (**a**) and hoop house (**b**) under three different N rates.

## Abbreviations

N	nitrogen
NUE	N use efficiency
NNI	N nutrition index
NUpE	N uptake efficiency
NUtE	N utilization efficiency
CP	crude protein
TP	total plant
N_a_	actual plant N concentration
N_c_	critical N concentration
