# Supplementary figures and images for: Opportunities for Increased Nitrogen Use Efficiency in Wheat for Forage Use
Source: Plants (Basel). 2020 Dec 9;9(12):1738. doi: 10.3390/plants9121738 (PMC7764361; doi:10.3390/plants9121738)

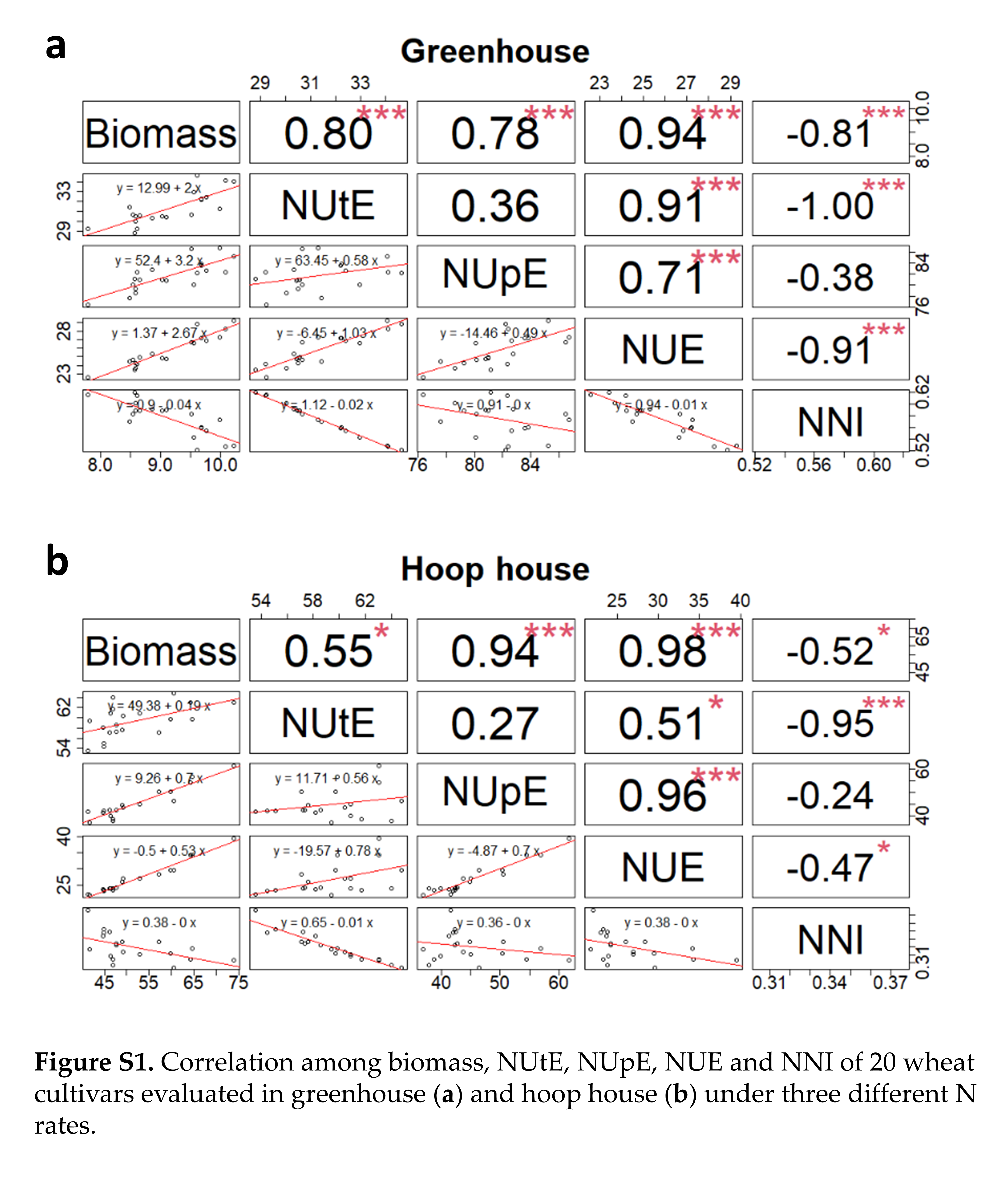

Supplement: Supplementary file 1 [file plants-09-01738-s001.zip › plants-1003796-supplementary.tif]
